# Calpain inhibition attenuates bleomycin-induced pulmonary fibrosis via switching the development of epithelial-mesenchymal transition

**DOI:** 10.1007/s00210-018-1499-z

**Published:** 2018-04-18

**Authors:** Yuan Liu, Bing Liu, Gu-qin Zhang, Jing-feng Zou, Meng-lin Zou, Zhen-shun Cheng

**Affiliations:** Department of Respiratory, Wuhan University Zhongnan Hospital, Wuhan, 430071 China

**Keywords:** Idiopathic pulmonary fibrosis, Epithelial-mesenchymal transition, Calpain, Differentiated embryonic chondrocyte-expressed gene 1

## Abstract

Calpains are intracellular calcium-dependent cysteine proteases, which cleave several substrates proteins, have been proven to play important roles in lung fibrosis. The aim of this study was to investigate the effects of calpain on bleomycin (BLM)-induced pulmonary fibrosis. A lung fibrosis mice model was established successfully by intraperitoneal injection of bleomycin. Calpeptin, a highly selective inhibitor of calpain activation, was administered three times weekly after bleomycin injection. Histological examination was used to assess the fibrosis. Quantitative-PCR and Western blotting were used to assess the development of epithelial-mesenchymal transition (EMT). We found calpeptin treatment decreased the BLM-induced EMT-associated markers, such as muscle actin (α-SMA) and collagen-I, while increased E-cadherin (E-cad). Calpeptin also suppressed the activation of transforming growth factor β1 (TGFβ1)-Smad2/3 signaling pathway, which plays crucial role in lung fibrosis and EMT. Furthermore, we found differentiated embryonic chondrocyte-expressed gene 1 (DEC1), an important transcription factor, was upregulated in both patients with idiopathic pulmonary fibrosis and in bleomycin-induced lung fibrosis. DEC1 was suppressed by calpeptin in bleomycin-induced mice model. Collectively, these findings indicated that calpeptin had a potential anti-fibrosis effect, which focus on the development of EMT.

**Electronic supplementary material:**

The online version of this article (10.1007/s00210-018-1499-z) contains supplementary material, which is available to authorized users.

## Introduction

Idiopathic pulmonary fibrosis (IPF) is a chronic lung disease of unknown cause with repeated acute lung injury causing progressive fibrosis, which finally leads to pulmonary architecture distortion and respiratory failure. The median time to death from diagnosis is 3 years (Wolters et al. 2014). Unfortunately, there are no widely accepted treatments against the progression of IPF.

Epithelial-mesenchymal transition (EMT) is a process when epithelial cells gradually transform into mesenchymal-like cells losing their epithelial functionality and characteristics (Greenburg and Hay 1982), which plays essential roles in the pathogenesis of pulmonary fibrosis. During this process, epithelial cells lose some cell markers, such as E-cadherin (E-cad), and acquire the mesenchymal markers, such as α-smooth muscle actin (α-SMA) and collagen-I. This switch in cell differentiation and behavior is mediated by some growth factors such as transforming growth factor β1 (TGFβ1) (Chapman 2011), while also mediated by many key transcription factors, including Snail, Twist, Zinc-finger E-box binding 1 (ZEB1), and some basic helix-loop-helix (bHLH) transcription factors (De Craene and Berx 2013; Peinado et al. 2007). Differentiated embryonic chondrocyte-expressed gene 1 (DEC1), also called STRA13 in mouse and SHARP2 in rat, is a bHLH transcription factor that is involved in the regulation of cell growth and differentiation (Qian et al. 2012; Rossner et al. 1997). It has been reported that DEC1 played an important role in the regulation of the EMT in hepatocellular carcinoma (Murakami et al. 2017). Loss of DEC1 expression is an early event in the development of lung cancer (Giatromanolaki et al. 2003). However, there is little evidence about the role of DEC1 in pulmonary fibrosis.

Calpains are intracellular calcium-dependent cysteine proteases. Both μ and m calpain are heterodimers formed with a large subunit (80KDa) specific for each isoform and an identical small subunit (28KDa) named calpain-4 (Goll et al. 2003). Calpains are ubiquitously expressed in mammalian cells, which cleave several cytosolic, membrane or cytoskeleton-associated proteins and play an important role in apoptosis (Bertoli et al. 2009), cell survival (Demarchi et al. 2005), cytoskeletal remodeling, and cell motility (Glading et al. 2002). However, the roles of calpain in lung fibrosis and in the development of EMT need to be further investigated.

In this study, we found calpain inhibitor protected lung fibrosis from bleomycin and decreased the expression of bleomycin-induced EMT-associated markers, such as α-SMA and collagen-I, while increased the expression of E-cad. Calpeptin also decreased the activation of TGFβ1-Smad2/3 signaling pathway. Furthermore, we found for the first time that DEC1 was upregulated both in IPF patients and in bleomycin (BLM)-induced lung fibrosis, while it was downregulated by calpeptin in BLM-induced mice model. Collectively, these findings indicated that calpeptin has a potential anti-fibrosis effect, which focus on the development of EMT.

## Methods

### Materials and reagents

Bleomycin was obtained from HISUN (ZheJiang, China). The calpain inhibitor calpeptin was purchased from Calbiochem (San Diego, USA). Antibody against calpain-1, calpain-2, α-SMA, collagen-I, DEC1, TGFβ1, and GAPDH were purchased from Abcam (Cambridge, UK). Antibodies against E-cad and phosphorylated-Smad2 as well as phosphorylated-Smad3 were obtained from Cell Signaling Technology (Danvers, USA).

### Animal model

The study protocols in this study were approved by the Animal Care and Use Committee of Zhongnan Hospital at Wuhan University. Pulmonary fibrosis was induced by bleomycin as multiple-dosage regimen. Eight-week-old male C57BL/6 mice were housed under standard condition with free access to water and rodent laboratory food. Bleomycin dissolved in 100 μl saline solution (concentration, 5 mg/ml) was administered by intraperitoneal injection on day 1, 5, 8, 11, and 15 respectively. Control mice received intraperitoneal injection with 100 μl saline solution. Calpeptin diluted in DMSO and dissolved in 0.2 ml of distilled water (concentration, 0.2 mg/ml) were injected intraperitoneally three times weekly. Mice from bleomycin group and control group were treated with or without calpeptin. Mice were sacrificed at day 28 for histological analysis and molecular experiment.

### Histological analysis

The mice were euthanized by thoracotomy. The right lungs were removed and snap frozen in liquid nitrogen for preparation of homogenates, and the left lungs were filled with 4% PFA solution and fixed for 24 h. The fixed lungs were then sliced mid-sagittally and embedded in paraffin. The slides (4 μm) were stained with hematoxylin and eosin and Masson’s trichrome selectively for morphometric analysis. Masson’s trichrome staining was performed as an index of collagen deposition. An OLYMPUS DP50-CU digital camera and Image-Pro Plus software were used to analyze the slides.

### Immunohistochemical staining

Samples were fixed in 4% PFA and processed into serial paraffin sections using routine procedure. Tissue sections (5 μm) were deparaffinized and rehydrated in xylene, heated in EDTA buffer, treated with 3% H_2_O_2_ in methanol for 10 min, and blocked with 5% normal serum. Sections were incubated with primary antibodies at 4 °C overnight. After incubation with secondary antibodies for 50 min at room temperature and DAB chromogen, the sections were counterstained with hematoxylin.

### Quantitative RT-PCR

Total RNA was extracted with TRIzol (Invitrogen, Carlsbad, CA, USA) according to the manufacturer’s instruction. cDNA was synthesized using Prime Script RT Reagent Kit with gDNA Eraser (TaKaRa, Dalian, China) following the manufacturer’s instructions. Quantitative PCR was conducted using a standard protocol from the SYBR GREEN PCR Kit (Toyobo, Osaka, Japan). The primer sequences used in the study were described in Table 1. Relative mRNA levels of the target genes were normalized to GAPDH mRNA expression and analyzed by the 2^−ΔΔCt^ method.

**Table 1:** Sequence of the primers used in the study

Gene	Forward primer	Reverse primer
E-cadherin	5′-TTCTTCGGAGGAGAGCGG-3′	5′-CAATTTCATCGGGATTGGC-3′
α-SMA	5′-CTATGCCTCTGGACGCACAAC-3′	5′-CCCATCAGGCAACTCGTAACTC-3′
Collagen-I	5′-CATGTTCAGCTTTGTGGACCT-3′	5′-GCAGCTGACTTCAGGGATGA-3′
DEC1	5′-GACAGTGGCTATGGAGGAGAATC-3′	5′-GTAGGCAGTCGCTGAAGGTG-3′
GAPDH	5′-TGGAAGGGTGGAGCCAAAAG-3′	5′-AGTCTTCTGGGTGGCAGTGAT-3′

### Western blot analysis

The lung tissues were homogenized in RIPA buffer and mixed with Western blot sample buffer. The lysates (40 μg protein) were denatured and electrophoresed on SDS-PAGE gel. Separated proteins were electrotransferred to PVDF membranes, blocked with 5% non-fat milk for 1 h, and then incubated with antibodies against calpain-1, E-cad (dilution 1:1500 for each), calpain-2, collagen-I, DEC1, p-Smad2, p-Smad3 (dilution 1:1000 for each), α-SMA, GAPDH (dilution 1:10000 for each), and TGFβ1 (dilution 1:500) at 4 °C overnight and then washed with TBST, and incubated with HRP-Goat anti Rabbit (ASPEN, AS1107) at room temperature for 1 h. Following three washes in TBST, bands were visualized by western electrochemiluminescence (ECL) kit (Tanon, Shanghai, China) and then exposed to X-ray film. The band densities were quantified using the AlphaEaseFC image analyzer system (Alpha Innotech, Inc., CA, USA), and the results were expressed as ratio of band density to total GAPDH.

### Statistical analysis

All data were analyzed with SPSS (version 17.0), and data were expressed as means ± SD. One-way ANOVA was used for multiple comparisons. Student’s *t* test was used for pairwise comparisons between two groups. *p* < 0.05 was considered statistically significant.

## Results

1. Calpain was upregulated in BLM-induced lung fibrosis.

**Fig. 1:**
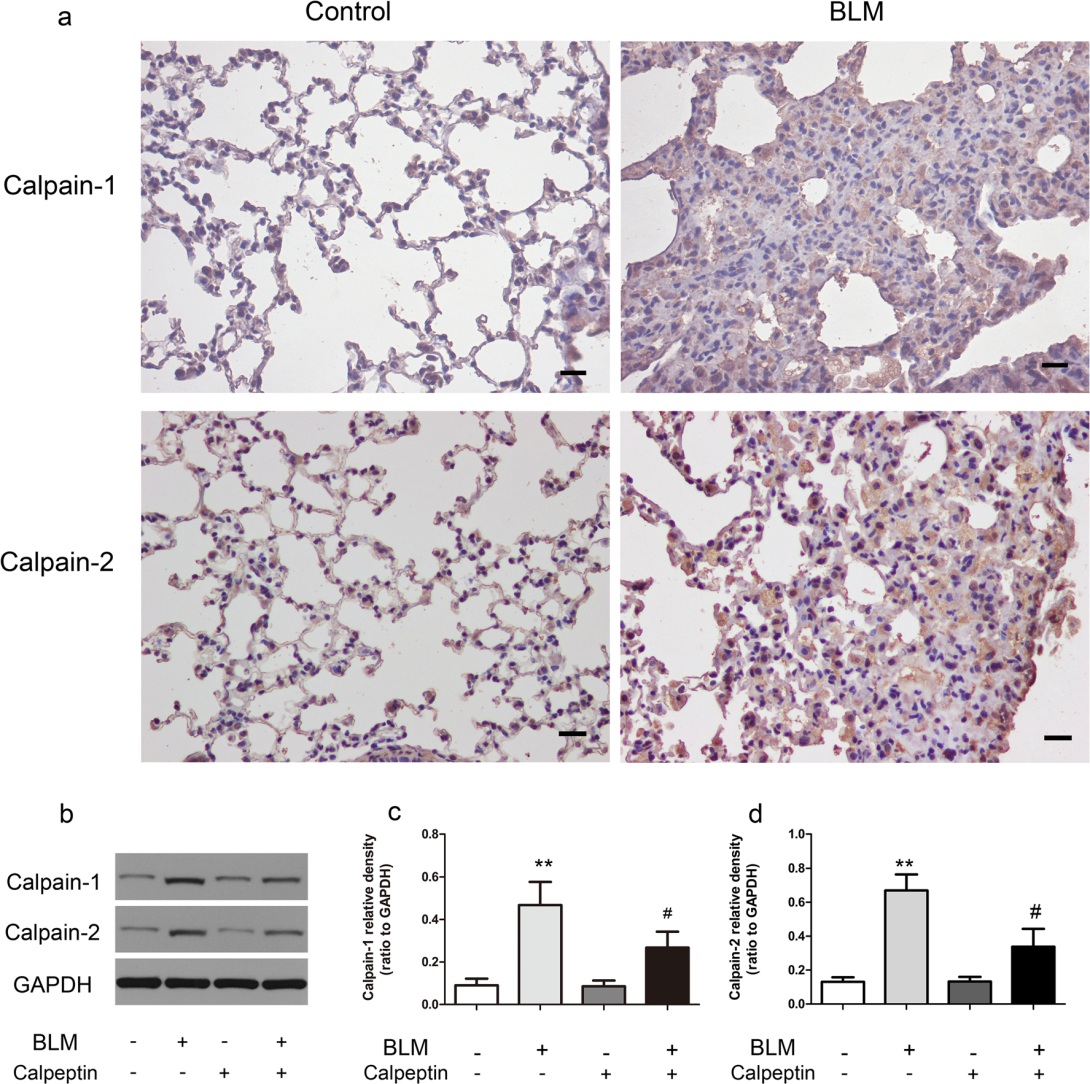
The expression of calpain-1 and calpain-2 in the lung tissue of BLM mice. Lungs from control mice and BLM-induced mice were taken for immunohistochemistry and western blotting. **a** Representative immunohistochemistry for calpain-1 and calpain-2 showed the expressions in bleomycin mice were significantly higher than in controls. *n* = 5; Scale bar: 200 μm. **b** Representative immunoblots showed the upregulation of calpain-1 and calpain-2 induced by BLM were decreased by calpeptin. **c**–**d** Changes in calpain-1 and calpain-2 quantified by scanning densitometry. Results were expressed as mean ± SD; *n* = 5 **p < 0.01 compared with controls; ^#^*p* < 0.05 compared with BLM group

To investigate the role of calpain in lung fibrosis, we took advantage of calpeptin, an inhibitor of calpain. We exposed C57BL/6 mice to bleomycin or saline as described in methods. Inmmunohistochemistry showed that expressions of calpain-1, calpain-2 in BLM-treated lungs were higher than in control lungs (Fig. 1a). Likewise, the protein levels of calpain-1, calpain-2 were increased, suggesting that BLM-induced calpain activation in lung fibrosis. However, the protein levels of calpain-1, calpain-2 were much lower in the lungs of calpeptin mice than in control mice (Fig. 1b–d), indicating that calpeptin inhibits calpain in vivo effectively.

2. Calpeptin protected lung fibrosis in mice from BLM

**Fig. 2:**
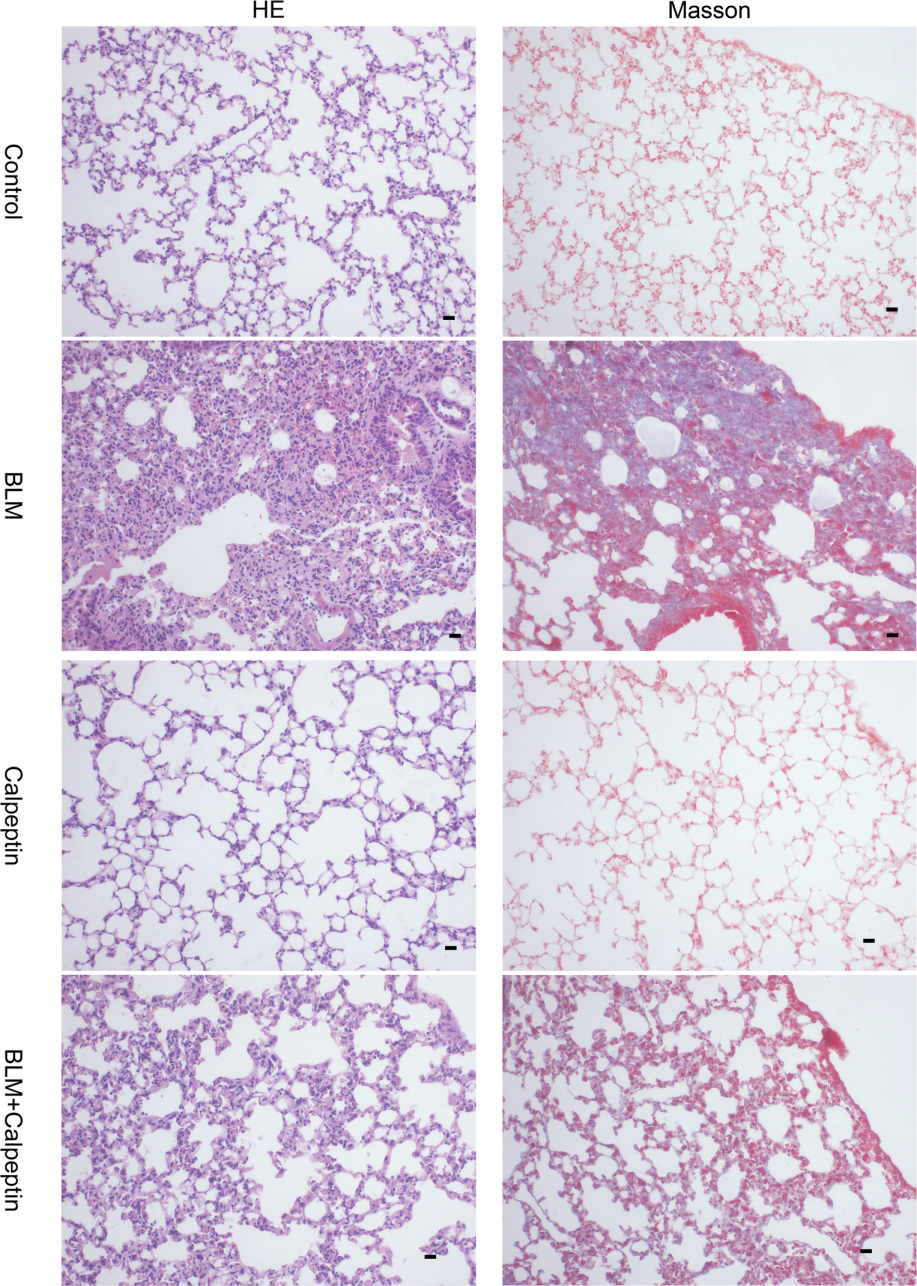
Morphological changes of calpain on BLM-induced lung fibrosis. Mice from BLM group and control group were treated with or without calpeptin. Hematoxylin-eosin staining and Masson’s trichrome staining were used on day 28 after BLM administration. Hematoxylin-eosin staining and Masson’s trichrome showed histologic changes of diffuse alveolar damage including edematous alveolar septa and deposition of collagen. *n* = 5; Scale bar: 200 μm.

The effects of calpeptin on BLM-induced lung injury and fibrosis were examined by morphological assess. Hematoxylin-eosin staining showed lung alveolar architecture damage and apparent increases in interalveolar septal thickness. Masson’s trichrome staining is an effective approach to assess the deposition of collagen, which appears to be blue. Abnormal collagen deposition was observed in BLM-treated mice, while the extent and intensity of the injury were less after calpeptin treatment (Fig. 2). The results suggested that calpeptin could protect pulmonary fibrosis from BLM.

3. Calpeptin suppressed the development of EMT in BLM-induced lung fibrosis.

**Fig. 3:**
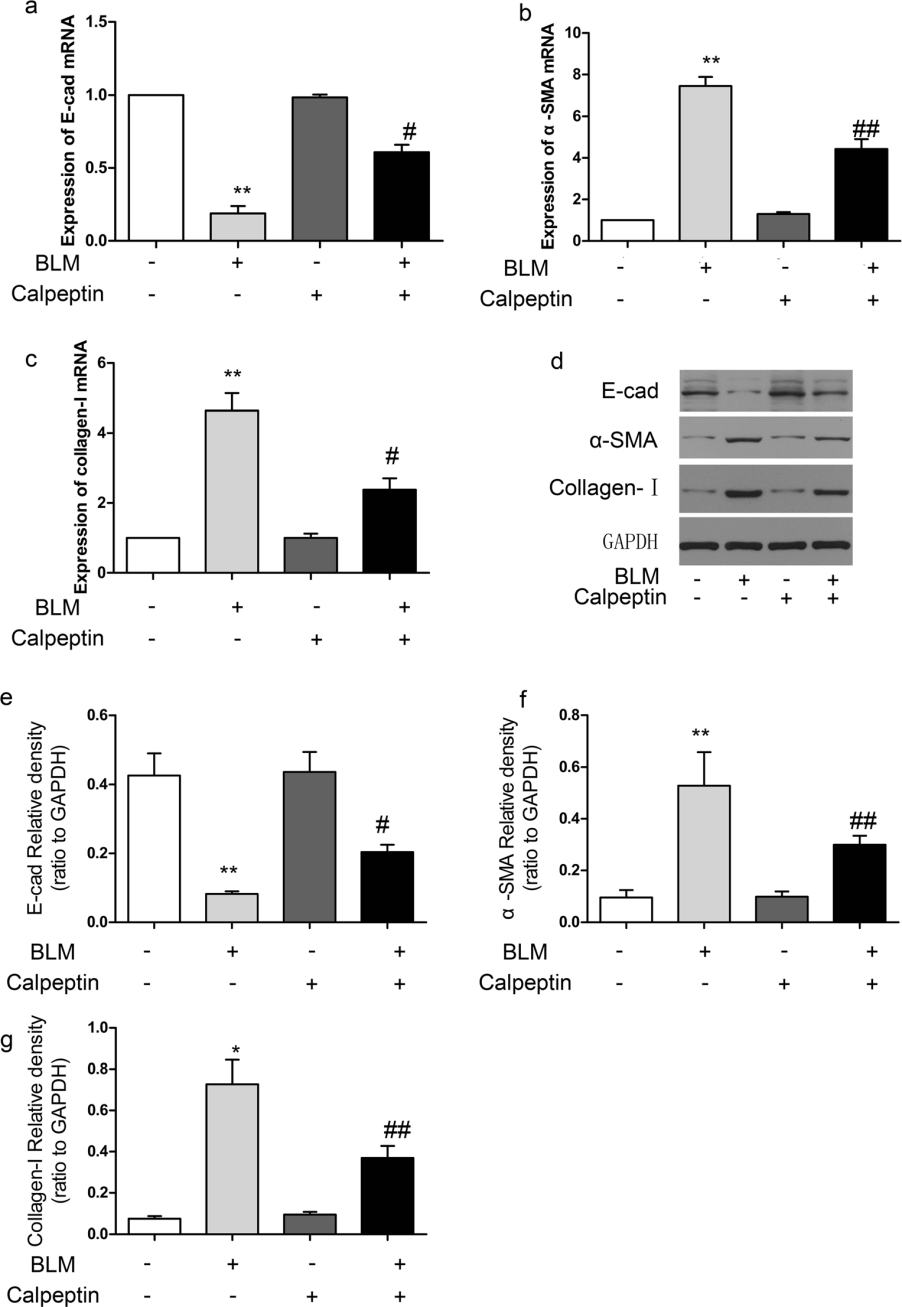
The effects of calpeptin on EMT process in BLM-induced lung fibrosis. BLM mice and control mice were treated with or without calpeptin. **a**–**c** RT-PCR analysis of E-cad, α-SMA, and collagen-I mRNA expression. **d** Representative immunoblots for E-cad, α-SMA, and collagen-I. **e**–**g** Changes in E-cad, α-SMA, and collagen-I quantified by scanning densitometry. Results were expressed as mean ± SD, *n* = 5, *p < 0.05 compared with controls; **p < 0.01 compared with controls; ^#^*p* < 0.05 compared with BLM group; ^##^*p* < 0.01 compared with BLM group

EMT is a major source of pathogenic fibroblasts during pulmonary fibrogenesis (Chapman 2011), which plays an important role in the pathogenesis of pulmonary fibrosis. We further examined the effects of calpeptin on EMT-associated markers. The decreased expression of lung alveolar epithelial marker E-cad and increased expression of mesenchymal marker α-SMA and collagen-I were observed in the lungs of BLM-treated mice. Furthermore, mice treated with BLM together with calpeptin attenuated downregulation of E-cad expression and upregulation of α-SMA and collagen-I expression compared with mice treated with BLM only (Fig. 3a–c). In addition, similar results were confirmed by western blotting (Fig. 3d–g). These results suggested that calpeptin may reduce the development of EMT in BLM-induced lung fibrosis.

4. Calpeptin suppressed TGFβ1-Smad2/3 signaling pathway in BLM-treated mice.

**Fig. 4:**
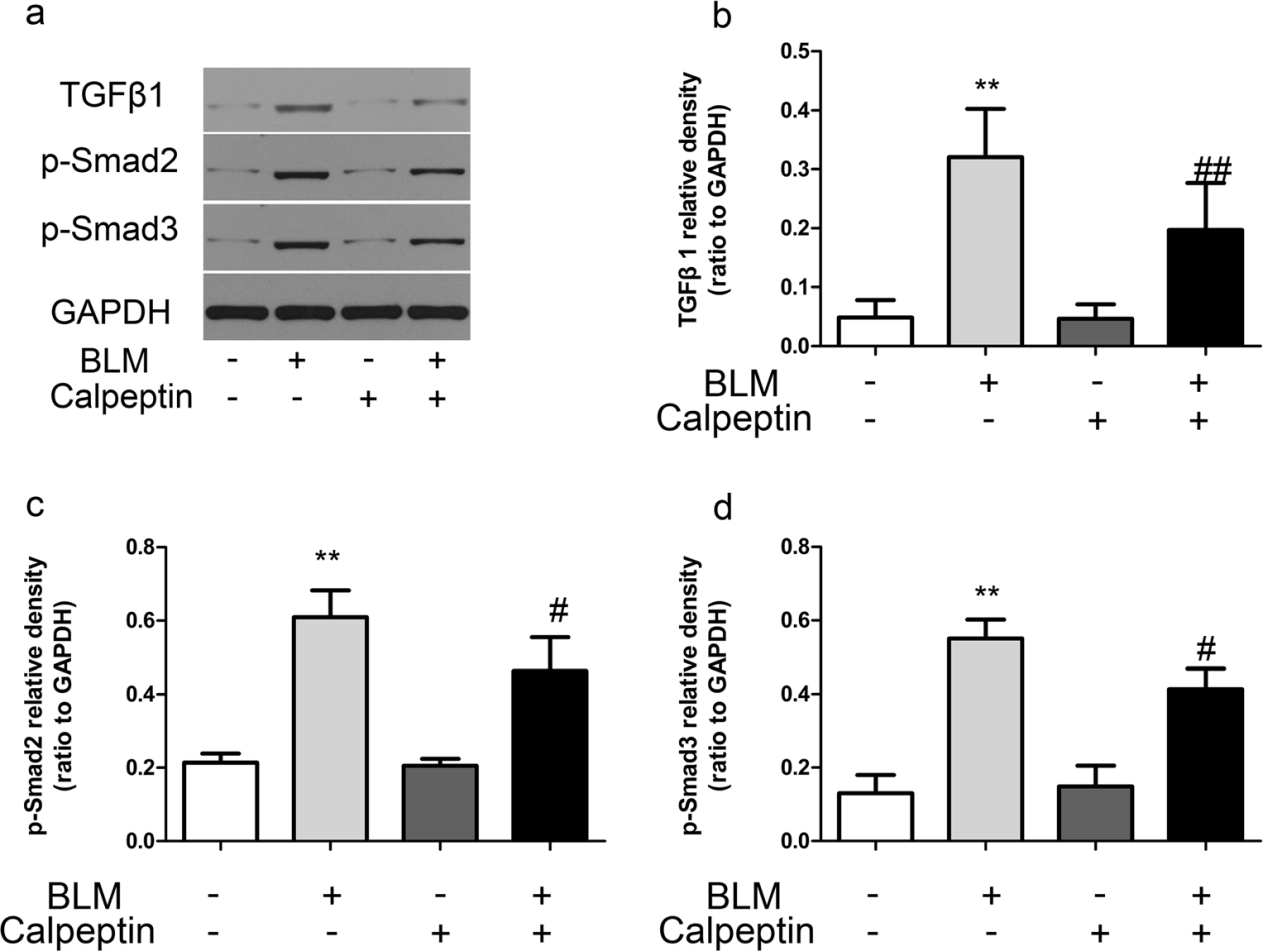
The effects of calpeptin on TGFβ1-Smad2/3 signaling pathway in BLM-induced lung fibrosis. BLM mice and control mice were treated with or without calpeptin. **a** Representative immunoblots for TGFβ1, p-Smad2, and p-Smad3. **b**–**c** Changes in TGFβ1, p-Smad2, and p-Smad3 quantified by scanning densitometry. Results were expressed as mean ± SD, *n* = 5, **p < 0.01 compared with controls; ^#^*p* < 0.05 compared with BLM group; ^##^*p* < 0.01 compared with BLM group

TGFβ1 is a “master switch” in the induction of EMT during the process of fibrosis in lungs. We further investigated the effect of calpeptin on the expression of TGFβ1 in lung fibrosis. TGFβ1 expression increased in BLM-induced mice, while the upregulation was suppressed by calpeptin. Since Smad2/3 phosphorylation is the major downstream regulator of TGFβ1 signal transduction, we examined Smad2 and Smad3 activation. The phosphorylation of Smad2 and Smad3 were identified in the lungs of belomycin mice, while they were inhibited by calpeption (Fig. 4). The results implied calpeptin may regulate the induction of EMT via TGFβ1-Smad2/3 signaling pathway.

5. DEC1 was upregulated in patients with IPF.

**Fig. 5:**
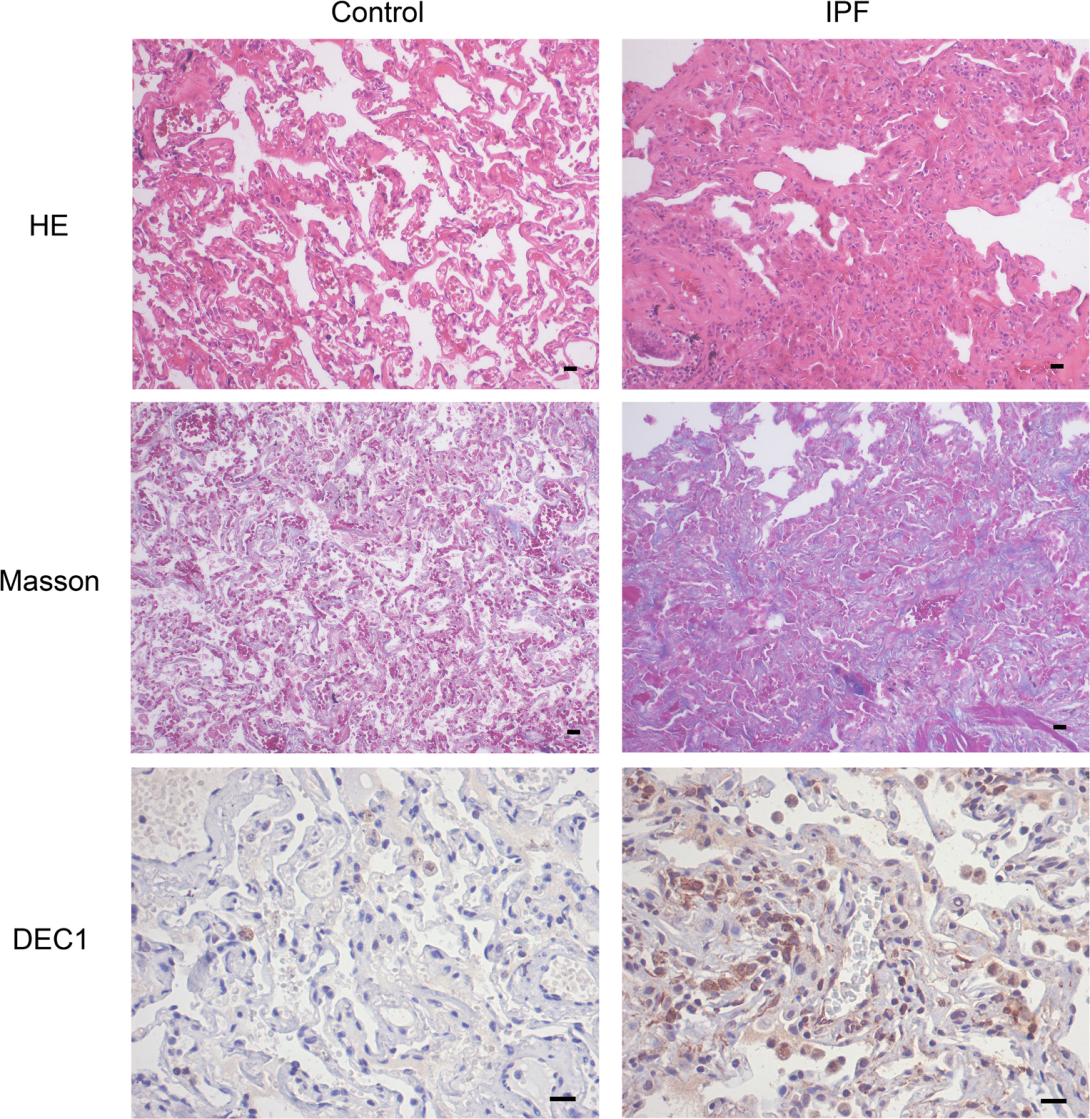
The expression of DEC1 in the lung tissue of IPF patients. Normal lungs and fibrosis lungs from patients were taken for hematoxylin-eosin staining, Masson’s trichrome, and immunohistochemistry. **a** Representative hematoxylin-eosin staining and Masson’s trichrome showed architectural destruction and the presence of dense acellular collagen. **b** Immunohistochemistry for DEC1 showed the expressions in IPF patients were significantly higher than in controls. *n* = 5; Scale bar: 200 μm

DEC1 belongs to the bHLH family of transcription factors and is able to regulate EMT in multiple carcinoma cells (Bi et al. 2015; Wu et al. 2012). However, whether DEC1 is involved in the pathogenesis of lung fibrosis has not been determined. Fibrosis lung tissue specimens were obtained from five patients with IPF (Raghu et al. 2015) who performed thoracic surgery at Zhongnan Hospital of Wuhan University. The clinical and pathological features were described in Table 2. Two normal lung specimens from resection of cancer were used as controls. Morphologically, the normal alveolar architecture was lost and replaced by areas with deposition of collagen fiber in IPF patients (Fig. 5a–b). The expression of DEC1was higher in the lung tissues of IPF patients than in controls (Fig. 5c). The findings indicated that DEC1 may play potential roles in IPF.

**Table 2:** The clinical and pathological features of study subjects

Case	Age	Sex	Clinical presentation	Smoking	Comorbidities	HRCT	Histology
1	45	M	Cough, finger clubbing	Yes	–	UIP	UIP
2	65	M	Cough, chest congestion	No	Respiratory failure	UIP	UIP
3	72	F	Cough, dyspnea	No	Hypertension	UIP	–
4	70	M	Cough, wheeze	Yes	Pneumonia	UIP	–
5	68	M	Cough, dyspnea	Yes	Pneumonia	UIP	UIP

*M*: male; *F*: female; *UIP*: usual interstitial pneumonia

6. Calpeptin suppressed DEC1 expression in BLM-induced lung fibrosis.

**Fig. 6:**
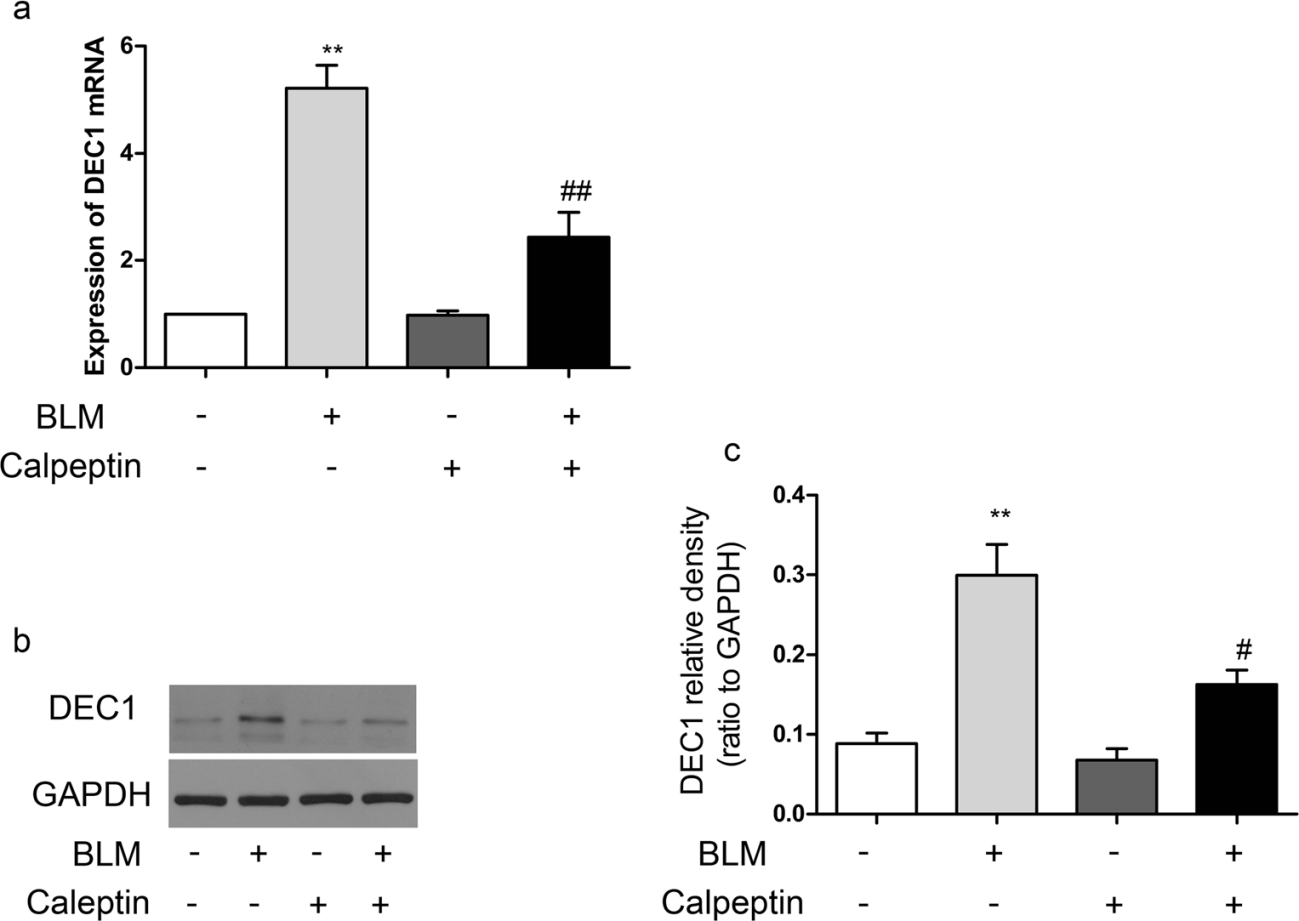
The relationship between calpain and DEC1 in BLM-induced lung fibrosis. BLM mice and control mice were treated with or without calpeptin. **a** RT-PCR analysis of DEC1 mRNA expression. **b** Representative immunoblot for DEC1. **c** Changes in DEC1 quantified by scanning densitometry. Results were expressed as mean ± SD, *n* = 5, **p < 0.01 compared with controls; ^#^*p* < 0.05 compared with BLM group; ^##^*p* < 0.01 compared with BLM group

To further investigate the role of DEC1 in the development of lung fibrosis. We examined the expression of DEC1 in BLM-induced lung fibrosis. Inmmunohistochemistry showed that expressions of DEC1 in BLM-treated lungs were higher than in the controls (Supplementary Fig. 1). Furthermore, DEC1 mRNA increased dramatically in the lungs of BLM mice compared to the controls; however, the upregulation was suppressed by calpeptin (Fig. 6a). Similarly, the changes of protein levels were confirmed by western blotting (Fig. 6b–c). The results indicated that calpeptin suppressed DEC1 in BLM-induced lung fibrosis.

## Discussion

IPF is a devastating lung disease whose incidence and prevalence increase markedly with aging (Nalysnyk et al. 2012). Respiratory failure is a major cause of death for individuals suffering from the disease; therefore, identifying the mechanism is important to limit the death. In the present study, we investigated the role of calpain in bleomycin-induced lung fibrosis. We found that calpeptin, an inhibitor of calpain, protected the lungs from bleomycin. We also found calpeptin suppressed the development of EMT directly via decreasing the expression of α-SMA and collagen-I, while increasing the expression of E-cad. Furthermore, we showed calpeption inhibited the induction of EMT via TGFβ1-Smad2/3 signaling pathway as well as suppressing the expression of DEC1.

We established lung fibrosis mice model successfully and found that calpain inhibition could protect lung fibrosis from bleomycin. The antineoplastic antibiotic, bleomycin, is widely used in cancer treatment and can cause interstitial lung disease in humans. BLM-induced lung fibrosis model is one of the earliest and still the most widely used animal model for understanding lung fibrosis. BLM instillation involves the predilection of dose-dependent fibrosis lung abnormalities (Snider et al. 1978). Single dose of BLM induce subchronic lesions, but more lasting fibrosis can result from repeated drug dosing (Chua et al. 2005). Therefore, we chose repetitive intraperitoneal BLM-induced mice to investigate the effect of calpain in pulmonary fibrosis. Calpains are a unique group of intracellular cysteine proteases found in almost eukaryotes and a few bacteria. Calpain-1 and calpain-2 were two major isoforms of conventional calpains, which were inhibited by calpeptin efficiently. We found that calpeptin protected lung fibrosis from BLM, which is consistent with a previous report by Tata and colleagues (Tabata et al. 2010).

We further investigate the mechanism of calpeptin inhibition in lung fibrosis. We indicated that calpeptin could suppress the induction of EMT through regulating associated cell markers (Fig. 3). E-cad is the classical epithelial marker that plays important role in epithelial cell adhesion, which works as a classical epithelial cell marker. α-SMA is the most commonly used molecular marker for myofibroblasts. α-SMA constitutes the major component of contractile stress fibers that are connected to transmembrane integrins at focal adhesions. The regulation of EMT centers on the transcriptional suppression of E-cad and the increase of α-SMA and collagen-I. Calpain may play a critical role in the process of EMT via its effects on cell motility and actin network remodeling, perhaps though cleavage of actin-associated cytoskeletal proteins (Storr et al. 2011). Initial research found that calpain localized to integrin-associated focal adhesion structures and directly cleaved the focal adhesion kinase (FAK) (Cuevas et al. 2003), which regulates cell-substrate attachment and cell motility. Furthermore, FAK is a specific substrate of calpains, which can be truncated into low molecular weight forms via activation of calpain (Perrin and Huttenlocher 2002).

Our study also found that calpeptin could suppress TGFβ1-Smad2/3 signaling pathway in lung fibrosis. TGFβ1 functions are crucial in fibrosis (Nanthakumar et al. 2015). Given that TGFβ1 as a “master switch” in the development of EMT during the process of lung fibrosis, we explored whether calpeptin inhibited the expression of TGFβ1. We found that calpeptin suppressed the levels of TGFβ1 and phosphorylation of Smad2/3, suggested calpain may be an up-stream of TGFβ1. Consistently, previous studies have showed that calpain cleaves and activates intracellular latent TGFβ1 and initiates the TGFβ1 pathway (Gressner et al. 2008; Ma et al. 2011). Liqun J and colleagues showed that overexpression of calpain-1 induced TGFβ1-Smad signaling pathway in age-associated central arterial wall stiffness (Jiang et al. 2012).

Furthermore, we found that DEC1 involved in the process of lung fibrosis. DEC1 functions as a transcription repressor by directly blinding to classic E-box in a highly specific manner. E-box is ubiquitously expressed in the promoter of target genes, including E-cadherin, occludins, and claudins (Ikenouchi et al. 2003). DEC1 directly regulate the target genes involved in many cancer cells (Bi et al. 2015; Seino et al. 2015). There are some similarities between carcinoma and IPF, so we examined whether DEC1 is involved in pulmonary fibrosis. We found for the first time that DEC1 was up-regulation both in lung tissues of IPF patients (Fig. 5) and in BLM mice (Supplementary Fig. 1). More importantly, we showed that DEC1 was suppressed by calpeptin not only in mRNA level but also in protein level. Given that calpain have some intracellular substrates among which are transcription factors, our finding was somewhat surprising, but not unexpected. One study has showed that transcription factors and calpain system were involved in squamous cell carcimoma metastasis (Spirina et al. 2013). Our observation may be attributable to the novel substrates of calpain in lung fibrosis. Other study indicated that DEC1 is a downstream target of TGFβ1 in EMT (Zawel et al. 2002). However, the mechanism how calpain regulate DEC1 needs to be addressed. And the detail mechanism of DEC1 in the induction of EMT in lung fibrosis also needs to be investigated in the future.

## Conclusions

In conclusion, this study indicated that calpain inhibition protected the lung fibrosis from bleomycin. The process is through the suppression of EMT and TGFβ1-Smad2/3 signaling pathway. DEC1 is proved to be participated in the process of lung fibrosis for the first time, which might be a key transcription factor in calpeptin-mediated inhibition of lung fibrosis.

## Electronic supplementary material

Supplementary Fig. 1 **The expression of calpain-1 and calpain-2 in the lung tissue of BLM mice.** Lungs from control mice and BLM-induced mice were taken for immunohistochemtry. Representative immunohistochemtry for DEC1 showed the expressions in bleomycin mice were significantly higher than in controls. *n*=5; Scale bar: 200μm. (GIF 195 kb)

High Resolution Image (TIFF 10612 kb)
