# Supplementary figures and images for: Calpain inhibition attenuates bleomycin-induced pulmonary fibrosis via switching the development of epithelial-mesenchymal transition
Source: Naunyn Schmiedebergs Arch Pharmacol. 2018 Apr 18;391(7):695–704. doi: 10.1007/s00210-018-1499-z (PMC5994212; doi:10.1007/s00210-018-1499-z)

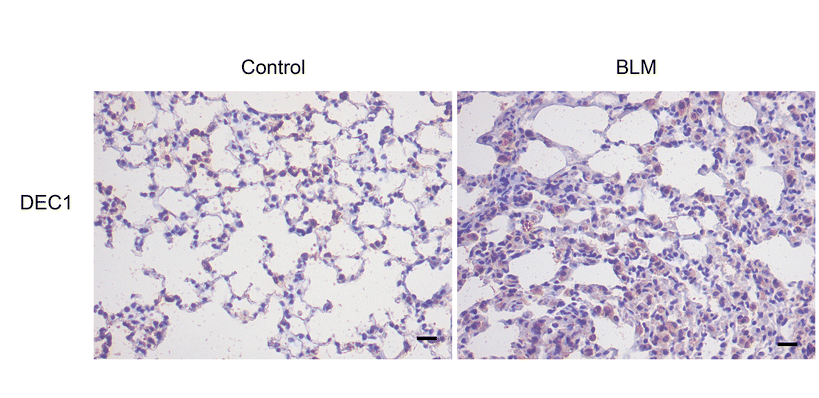

Supplement: Supplementary file 1 — The expression of calpain-1 and calpain-2 in the lung tissue of BLM mice. Lungs from control mice and BLM-induced mice were taken for immunohistochemtry. Representative immunohistochemtry for DEC1 showed the expressions in bleomycin mice were significantly higher than in controls. n=5; Scale bar: 200μm. (GIF 195 kb) [file 210_2018_1499_Fig7_ESM.gif]

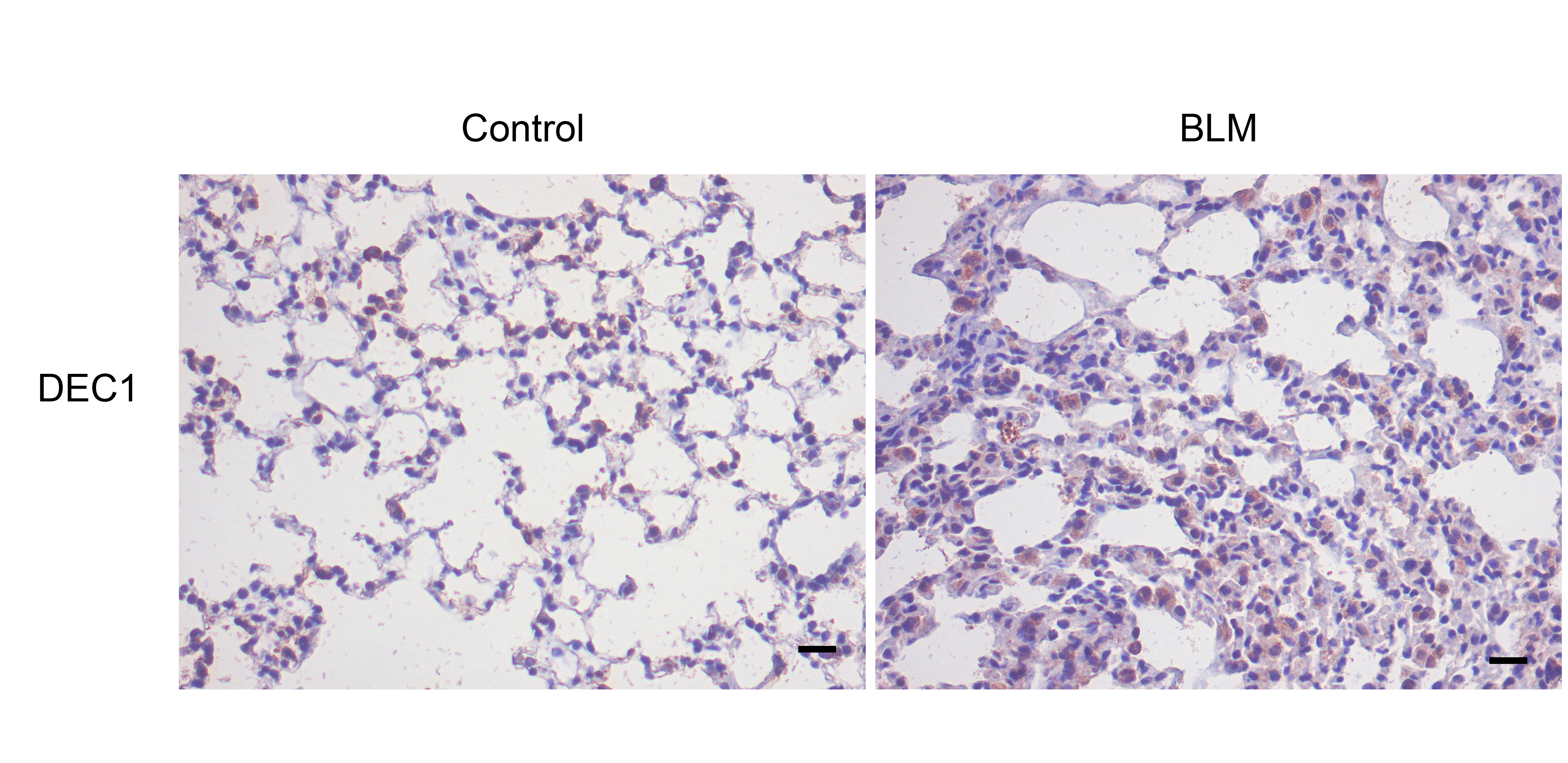

Supplement: Supplementary file 2 — High Resolution Image (TIFF 10612 kb) [file 210_2018_1499_MOESM1_ESM.tif]
